# Retraction: Balancing mitochondrial dynamics via increasing mitochondrial fusion attenuates infarct size and left ventricular dysfunction in rats with cardiac ischemia/reperfusion injury

**DOI:** 10.1042/CS20190014_RET

**Published:** 2025-06-23

This article “Balancing mitochondrial dynamics via increasing mitochondrial fusion attenuates infarct size and left ventricular dysfunction in rats with cardiac ischemia/reperfusion injury” DOI:10.1042/cs20190014 Maneechote et al., Clin Sci (Lond) (2019) 133 (3): 497–513 is being retracted from *Clinical Science* at the request of the Editor-in-Chief and the Editorial Board. This follows receipt of a notification from a reader, alerting the Editorial Board that Figure 5 appears to contain signs of digital splicing, and two protein bands in the Cleaved Caspase 3 panel have a similar appearance.

The Editorial Office identified further concerns in the Western blots in this paper. Similarities were noted between the two “During-ischemia” protein bands in Figure 3B pCx43(ser368). Additionally, Figure 3B β-actin Sham band appears similar to Figure 5 Control band, and Figure 3B β-actin Control band appears similar to Figure 5 Sham band.

The authors were contacted regarding the concerns. The authors state that the Western blot for the Sham group was added separately to Figure 5, as this condition was not included in the original experiments. The authors provided original data for the Western blots; however, this has not resolved the issues identified. The journal no longer has confidence in the reliability or validity of the results presented in this paper, and given the extent of the issues raised, the Editorial Board stand by the decision to retract the article. The authors disagree to the retraction.

